# An Anti-Fat

**Published:** 1880-01

**Authors:** 


### SEA-WEED AS AN "ANTI-FAT."

The use of the variety of sea-weed botanically known as *Fucus vesiculosus* for reducing obesity has obtained recognition in professional circles abroad. Dr. Fairbank writes to the *British Medical Journal* as follows: "More than fifteen years ago, I gave some of the extract in pills (four grains three times a day) to a very corpulent lady, who in three months lost three stone in weight without any change of diet. Since then, I have frequently given it for reducing weight depending on the accumulation of adipose tissue, and have never found it to fail. The solid extract can be easily made into four-grain pills, which must, however, be kept in a stoppered bottle, as they readily absorb moisture from the air. I may say that a patient, who has lately been taking it as an antifat, and who always suffered very much from rheumatic pains about the body, has been entirely free from such trouble while she has been taking the extract, a fact which she quite independently noted."

